# Systematic Review on the Impact of Conditional Cash Transfers on Child Health Service Utilisation and Child Health in Sub-Saharan Africa

**DOI:** 10.3389/fpubh.2021.643621

**Published:** 2021-07-14

**Authors:** Chukwuemeka Onwuchekwa, Kristien Verdonck, Bruno Marchal

**Affiliations:** Department of Public Health, Institute of Tropical Medicine, Antwerp, Belgium

**Keywords:** conditional cash transfer, child, health service utilisation, health status, sub-Saharan Africa

## Abstract

**Background:** Conditional cash transfers (CCTs) are interventions which provide assistance in the form of cash to specific vulnerable groups on the condition that they meet pre-defined requirements. The impact of conditional cash transfers on children's access to health services and on their overall health has not been established in sub-Saharan Africa.

**Method:** We conducted a systematic review aimed at summarising the available information on the impact of conditional cash transfers on health service utilisation and child health in sub-Saharan Africa. We searched databases for peer-reviewed articles, websites of organisations involved in implementing conditional cash transfer programmes, and Google scholar to identify grey literature. Records were selected based on predefined eligibility criteria which were drawn from a programme impact framework. Records were eligible if one of the following outcomes was evaluated: health services utilisation, immunisation coverage, growth monitoring, anthropometry, illness reported, and mortality. Other records which reported on important intermediate outcomes or described mechanisms significantly contributing to impact were also included in the review. Data items were extracted from eligible records into an extraction form based on predefined data items. Study quality indicators were also extracted into a quality assessment form.

**Results:** Thematic narrative synthesis was conducted using data from nine included records. The review included five cluster randomised evaluations, one quasi-experimental clustered study, one randomised trial at the individual level, one mixed-method study and one purely qualitative study. There was insufficient evidence of an impact of conditional cash transfers on health service utilisation. There was also not enough evidence of an impact on nutritional status. No impact was observed on health status based on illness reports, nor on immunisation rates. None of the included records evaluated the impact on childhood mortality.

**Conclusions:** The findings of this review suggest that a positive impact may be observed in health service utilisation and nutrition, however, this may not translate into improved child health. Further research is needed to understand the mechanisms and pathways by which these interventions work, explore the effect of contextual factors on their impact, and assess their cost implication especially within resource-constrained settings.

## Introduction

Cash transfers are defined as the provision of assistance in the form of cash with the objective of increasing the household's real income (1). Conditional cash transfers (CCTs) provide monetary transfers to targeted populations (usually poor, vulnerable and underserved persons) as long as they adhere to specific programme requirements or conditions–for example children attend regular clinics and receive immunisations, or pregnant women attend regular antenatal clinics and deliver in a health facility (1, 2). Cash transfers are aimed at relieving some of the financial constraints poor households face in accessing essential services like health care.

Since their introduction in Latin America in the 1990s, several evaluations have been conducted to assess the impact of CCTs on health service utilisation and health. Much of the evidence on the impact of CCTs comes from earlier programs in Latin America (1), the Caribbean (3) and parts of Asia (4, 5). These early impact evaluations suggest that CCTs, may have a positive impact on the diet of children from poor households and improve their nutritional status (2, 5–10), on attendance at routine clinical visits (7, 9, 11–14), on the uptake of routine childhood immunisation (4, 9, 11, 13), and, in the long term, on child health (7, 15). However, these findings have not been consistent across studies, and some positive impacts were not sustained after longer periods of programme implementation (9, 16, 17). Moreover, the effect of CCTs on child health and health service utilisation has not been established in sub-Saharan Africa where access to services is often inadequate and child health indicators remain poor.

This review sought to critically assess and summarise the available information on the impact of conditional cash transfers (CCTs) on child health and health service utilisation in sub-Saharan Africa. Specifically, we aim to summarise the evidence on the impact of CCT on routine health visits, uptake of immunisation services, child nutrition state and frequency of reported illness.

## Methodology

### Study Design

This is a review of published literature, programme reports and working papers detailing the impact of conditional cash transfers on child health and health service utilisation in sub-Saharan Africa. We started with a programme impact pathway as described by Leroy et al. (2), with elements from the work of de Groot et al. (18), to explore the pathways by which CCTs may impact on child health and health service utilisation (Supplementary Figure 1). Wherever possible, we adhered to the Preferred Reporting Items for Systematic reviews and Meta-Analysis (PRISMA) and the Systematic reviews Without Meta-analysis (SWiM) guidelines for reporting systematic reviews (19, 20). The research methods, including the search strategy, eligibility criteria and a preliminary plan for analysis and synthesis, were developed in advance of data extraction.

### Eligibility Criteria

The SPICE framework (settings, perspective, intervention, comparison and evaluation) was used to formulate the research question (21). The research question is further detailed in Table 1.

**Table 1:** Details of review question using the SPICE framework.

**Element**	**Description**
Setting	Any of the countries within sub-Saharan Africa where a conditional cash transfer intervention has been implemented
Perspective	Children <5 years of age
Intervention	Any intervention where cash is provided to individual or household on the condition that they fulfil specific health related conditions
Comparison	Comparison groups who benefit from CCT intervention and those who do not, populations before and after the CCT intervention
Evaluation	Impact on use of preventive and curative health services, nutritional status and health status

We identified records of studies on the impact of CCTs from any country within sub-Saharan Africa. Records of studies on unconditional cash transfers and in-kind transfers were excluded. We included studies reporting on the impact of CCT intervention on an indicator of health access or utilisation (for instance attendance at routine clinics or immunisation uptake), or an indicator of health or nutrition (for instance frequency of reported illness or anthropometric measurements) in children who are under 5 years of age.

For a study to be eligible, it had to include a formal comparison between beneficiaries and non-beneficiaries, using either a contemporary or a historical comparison group. We included primary studies with the following designs: cluster-randomised studies, randomised-controlled trials, before-and-after studies and interrupted time-series designs. No time or language restrictions were put in place.

### Information Sources

Records were identified following a systematic search of PubMed, EBSCO e-journal, EBSCO global health and African Index Medicus (AIM) using the search strategy detailed in Supplementary Table 1. The first database search was conducted on 29 February 2020 (PubMed) and the last search on 13 March 2020 (EBSCO e-journal). In addition, we searched the websites of the World Bank (https://openknowledge.worldbank.org) and the Institute for Fiscal Studies (https://www.ifs.org.uk) for relevant titles. Finally, we searched Google scholar for reports that may have been missed by previous searches.

### Search Strategy

The search strategy combined the following key themes of the research question:

- Conditional cash transfers
- Children under-five
- Sub-Saharan Africa

The search strategy was initially developed for PubMed and subsequently adapted to other indexed databases. The complete strategy for each search is included as Supplementary Table 1.

### Study Selection

The titles and abstract of records retrieved from the search were screened to identify potentially relevant records. For the records that were deemed eligible at this stage, the full-texts were obtained and screened. Records that met the eligibility criteria after the full-text screening were then included in the review. We also conducted a manual search of the reference lists of included records to identify additional records for inclusion into the review.

### Data Collection Process and Data Items

Data extraction was conducted using a pre-piloted extraction form developed in Excel containing relevant data items (Supplementary Table 2). The following specific data items were collected: first author name and affiliation, year of publication, article source; programme name, country, rural or urban setting, year intervention initiated, situation under which intervention was conducted (stable or unstable), cash transfer amount in USD, frequency of transfers, direct recipient, any concurrent supply-side interventions, and description of transfer conditions, baseline year, evaluation year, population evaluated, evaluation design, type of analysis, data collection technique, outcomes evaluated, and findings.

### Quality Assessment of Individual Studies

We developed a quality assessment instrument for this review, designed to capture the specific quality dimensions of impact studies (Supplementary Table 2). Our assessment instrument borrows elements from The National Heart, Lung and Blood Institute quality assessment tool for randomised interventions, and the Methodological index for non-randomised studies (MINORS) instrument (22). This assessment instrument was not designed to assign a “quality score” to studies, but to provide an overview of the quality assessment of each study. The elements and questions in the instrument are presented in Box 1 below. We also used the assessment tool developed by Walsh and Downe, to appraise the qualitative studies (23).

Box 1 **Elements of the quality assessment instrument used in the review.**

**Table:** 

**Element**	**Question asked**	
Study method	Was the study method appropriate to the question the study set out to answer?	
Sample size	Was the sample size measured prospectively? Was sample size justified?	
Outcome assessment	Were outcome assessors blinded to the allocation of the respondent?	
Randomisation	Did any event occur during conduct of the study that could have compromised randomisation? (include contamination)	
Outcome measurement	Were all outcomes measured in a valid or objective way?	
Analysis	Was the analysis appropriate? (include control for confounding, appropriate consideration for time trend)	
Adequate comparison	Were the groups comparable at baseline?	
Attrition or loss to follow-up	Was loss to follow-up or attrition significant? (more than 5%) Was loss to follow-up or attrition different between groups?	
Conflict of interest	Was there potential conflict of interest between authors or sponsors and the CCT implementing institution?	

### Data Presentation and Synthesis

Because context, study design, and outcome assessment varied across the included studies, we conducted a narrative synthesis and did not attempt to quantitatively pool the findings. We first describe the general characteristics and the quality of the included studies. Next, we present the key findings focusing on both intermediate and final outcomes.

### Ethical Considerations

The review did not directly involve human subjects and ethical concerns are therefore minimal. However, during the review process, due consideration was given to the ethical conduct of included studies (24).

## Results

### Summary of Eligible Records

**Figure 1:**
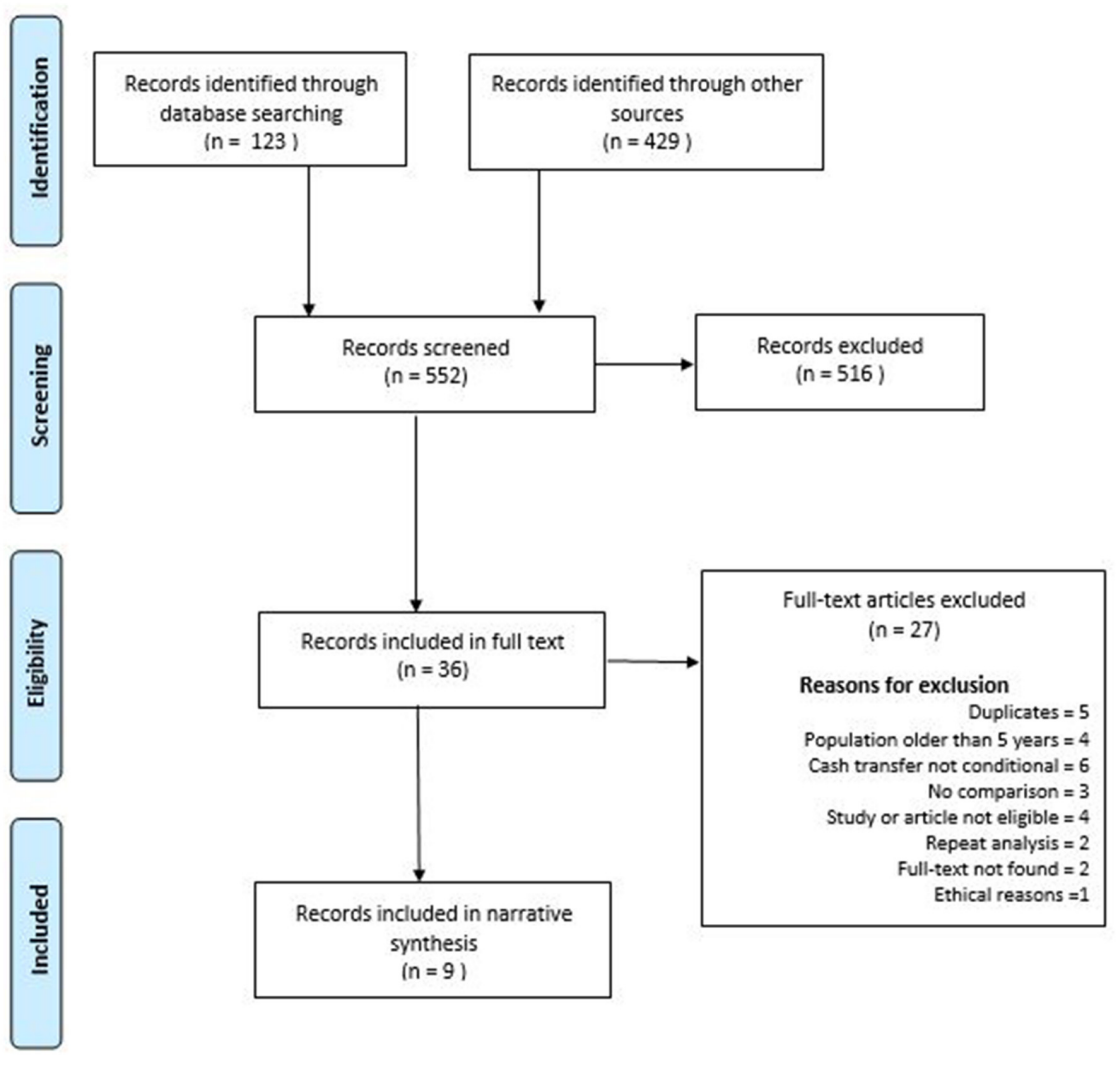
PRISMA flow chart showing the record selection process.

The search of indexed databases produced 123 records, while other sources produced 429 records. Nine of these records were included, describing eight conditional cash transfer programmes in seven sub-Sahara African countries implemented between 2008 and 2016. The record selection process is detailed in a PRISMA diagram (Figure 1).

### Study Characteristics

#### Description of Programmes

Table 2 describes the CCT programmes included in the review. Six programmes were pilot projects, one was a research project and one was an emergency programme (addressing a drought). All but one of the programmes were conducted in rural settings, which were considered poor or underserved (31).

**Table 2:** Description of programmes evaluated in the review.

**CCT programme**	**References**	**Country and setting**	**Situation, target**	**Cash transfer schedule**	Related conditions^*^	**Evaluation design**	**Type of analysis**	Follow-up period^**^	**Organisation(s) implementing CCT**
Manicaland HIV/STD prevention project	(23)	Zimbabwe Rural	Pilot, Vulnerable household in selected communities	$18 for household, $4 per child to max 3 children bimonthly	Up-to-date immunisation in children under 5 years Growth monitoring twice a year Parenting skill classes for family representative.	Matched cluster randomised experiment	Difference-in-Difference	12 months	Wellcome trust, World Bank, United Nations children's fund
Concern worldwide CCT programme	(25)	Niger, Rural	Emergency famine, Vulnerable households	$250 over 3 months to households	Attendance of health and nutrition classes by mothers	Quasi-experimental	Difference-in-Difference	3–6 months	Concern worldwide
SNACK-CNA programme^‡^	(26)	Mali, Rural	Pilot, Vulnerable households in selected communities	Approximately $96 over pregnancy up to 24 months (paid per condition completed $4/vaccination, $3 /Growth monitor)	Vaccination. Monthly growth monitoring visits	Cluster randomised experiment	Regression analysis	24 months	Global affairs Canada, World Food Programme and United Nations children's Funds
	(27)					Mixed methods	Descriptive Thematic analysis	Approximately 12 months	
SURE-P MCH^‡^	(28)	Nigeria, Rural	Pilot, Selected health facilities	$30 over pregnancy up to 6 weeks post-natal ($6 at first immunisation) to mothers	OPV-1 vaccination	Quasi-experimental	Time-series	12 months	SURE-P MCH, children investment fund foundation, Mckinsey & Company
Nahourio CTPP	(29)	Burkina Faso, Rural	Pilot, Vulnerable households in selected communities	$9.64 per child per year to households	Quarterly visits to health facility for growth monitoring	Cluster randomised experiment	Regression analysis	21 months	World Bank
TASAF	(30)	Tanzania, Rural	Pilot, Vulnerable households in selected communities	$14.5 ($12–36) bimonthly to households	Vaccination. Six visits to health facility for weight monitoring	Cluster randomised experiment	Regression analysis	22 months	World Bank
Not applicable^‡^	(31)	Democratic Republic of Congo, Urban	Research	Maximum $45 from pregnancy to 6 weeks post-natal^#^	HIV early infant diagnosis at 6 weeks	Individual randomised controlled experiment	Regression analysis (Intention-to-treat)	12 months	PEPFAR and the NIHCD
LEAP	(32)	Ghana, Rural	Pilot, Vulnerable households	$21–39 per month to households (plus health insurance)	Health check-ups	Qualitative methods	Thematic analysis	Approximately 24 months	Ghana Ministry of Gender, Children and Social Protection

*SNACK-CNA, Santé Nutritionnelle à Assise Communautaire à Kayes–cash for nutritional awareness; HIV, Human immunodeficiency virus; STD, sexually transmitted diseases; SURE-P MCH, Subsidy Reinvestment and Empowerment Programme Maternal and Child Health Project; CTTP, Cash Transfers Pilot Project; TASAF, Tanzania Social Action Fund; LEAP, Livelihood Empowerment Against Poverty; OPV-1, Oral Polio Vaccine first dose; PEPFAR, The President's Emergency Plan for AIDS Relief; NIHCD, National Institute of Health and Child Development*.

* *Describes conditions related to utilisation of child services*.

** *Period between initiation of intervention and final evaluation*.

‡ *CCTs initiated during pregnancy*.

# *CCTs targeting HIV prevention of mother-to-child services*.

The amount of cash given to beneficiaries varied between programmes (see Table 2), and was mostly received by adult women in the households.

#### Description of the Evaluation Designs

Seven of the included studies focused on the quantitative impact of the CCT programme, while two studies were of a qualitative nature.

##### Quantitative Evaluations

As detailed in Table 2, the study methodology of the quantitative studies varied considerably. Six were clustered studies with or without random assignment, and one was a randomised trial at the individual level. All clustered studies conducted baseline and follow-up surveys, with the follow-up surveys typically conducted 1–2 years after initiation of the CCT.

##### Qualitative Evaluations

The two qualitative studies included in this review used interviews with mothers or caregivers of children from CCT recipient households. Respondents were selected purposively in both evaluations. Data collection was by individual and/or group interviews using a semi-structured format; in both studies, only beneficiaries were interviewed. Respondent triangulation was done in both evaluations.

### Outcomes Measured in Evaluations

The studies included in this review reported on the following quantitative outcomes: immunisation rates (*n* = 3), health centre attendance (*n* = 3), illness or ill days preceding the survey (*n* = 3), nutritional status [*n* = 3, measured as weight, height, height-for-age z-score (HAZ), weight-for-age z-score (WAZ), weight-for-height z-score (WHZ), and mid-arm circumference (MUAC)], dietary intake (*n* = 1, measured as frequency and diversity from 24-h recall from care-givers, mothers' knowledge on health and nutrition (*n* = 1), and early infant intervention in HIV/AIDS (*n* = 1).

### Quality Assessment of Individual Studies

#### Quantitative Evaluations

Overall, all included quantitative studies had at least one significant quality concern. A key area of concern in many included studies was the absence of effective observer blinding. Potential conflict of interest was also identified in all included studies (Supplementary Table 2).

#### Qualitative Evaluations

The guidance by Walsh and Downe allows appraising qualitative studies based mainly on sample size determination, triangulation and reflexivity (33). Details of the quality assessment are presented in Supplementary Table 2. Overall, we identified gaps in the reporting of the justification on the sample-size and the potential influence of reflexivity in one of the two qualitative studies (32).

### Summary Findings of Individual Studies

The key findings of the included studies are summarised in Table 3 below.

**Table 3:** Summary of finding of evaluations included in the review.

**References**	**Programme**	**Study design**	**Age**	**Key findings**
Robertson et al. (23)	Manicaland HIV/STD prevention project, Zimbabwe	Matched cluster randomised study	0–59 months	No significant difference in up-to-date immunisation records
Bliss et al. (25)	concern worldwide CCT programme, Niger	Quasi-Experimental design	6–24 months	No difference in reported illness in preceding 15 days
				Significant difference in anthropometry (Weight, WHZ and MUAC)
				Significant increase in meal frequency and diversity in preceding 24 h
Adubra et al. (26)	SNACK–CNA, Mali	Cluster randomised study	12–42 months	No observed difference in immunisation rates
				No difference in number of routine growth monitoring visits in past year
				No difference in illness reports in preceding 15 days
				No difference in meal frequency and diversity in preceding 24 h
				No difference in HAZ
Okoli et al. (28)	SURE-P MCH, Nigeria	Quasi-experimental design	<6 weeks	No difference in OPV rate in intervention and control sites
Akresh et al. (29)	Nahourio CTPP, Burkina Faso	Factorial cluster randomised study	0–59 months	More routine clinic visit per year in CCT recipients
Evans and Hausladen (30)	TASAF, Tanzania	Cluster randomised study	0–23 months	No difference in number of health facility visits at midline
			0–59 months	No difference in anthropometry (Height, Weight, HAZ, WAZ, WHZ, MUAC).
			0–59 months	No significant difference in reported illness in preceding 4 weeks.
			Household level	Increase in dietary intake. No increase in consumption of harmful commodities
Yotebieng et al. (31)	Not applicable	Individual randomised control trial	<6 weeks	No significant difference in proportion of children receiving early infant diagnosis for HIV

*SNACK-CNA, Santé Nutritionnelle à Assise Communautaire à Kayes–cash for nutritional awareness; HIV, Human immunodeficiency virus; STD, sexually transmitted diseases; SURE-P MCH, Subsidy Reinvestment and Empowerment Programme Maternal and Child Health Project; TASAF, Tanzania Social Action Fund; WHZ, Weight-for-Height Z–score; MUAC, mid-upper arm circumference; HAZ, Height-for-Age Z-score; WAZ, Weight-for-Age Z-Score; CTTP, Cash Transfers Pilot Project*.

#### Synthesis of Findings

##### Effect on Dietary Intake

Two studies reported on the impact of a CCT programme on household dietary intake. The emergency CCT programme in Niger and the Tanzania Social Action Fund (TASAF) programme in Tanzania both showed an increase in consumption of most food groups among beneficiaries, most importantly in the consumption of proteins (25, 34).

##### Effect on Nutritional Outcomes

Three studies reported on nutritional outcomes, with only one showing a positive effect in CCT beneficiaries (Table 4). In the emergency CCT intervention in Niger, beneficiaries gained significantly more weight than controls (25).

**Table 4:** Effect of CCT on anthropometric measures.

**References**	**Study Design**	**Age (months)**	**Outcome**	**Baseline (CCT)**	**Baseline (control)**	**Effect size**
Bliss et al. (25)	Quasi-Experimental design	6–24	Weight—mean and sd (kg)	7.89 (1.00)	8.24 (1.00)	1.35 (*p* < 0.001)^a^
			WHZ—mean and sd	−1.5 (1.1)	−1.0 (1.1)	1.83 (*p* < 0.001)^a^
			MUAC—mean and sd (mm)	137 (8)	139 (9)	7.0 (*p* < 0.001)^a^
Adubra et al. (26)	Cluster randomised design	12–42	HAZ—mean and sd	−1.57 (1.23)	−1.40 (1.23)	0.03 (*p* = 0.75)^b^
			Prevalence of stunting (%)	35.6	29.5	0.87 (*p* = 0.32)^c^
Evans and Hausladen (30)	Cluster randomised design	0–59	Height—Mean (cm)	87.31 (combined)		0.53 (*p* >0.1)^b^
		0–59	Weight—Mean (kg)	12.16 (combined)		0.16 (*p* >0.1)^b^
		0–59	MUAC—Mean (mm)	155.81 (combined)		1.42 (*p* >0.1)^b^

*WHZ, Weight-for-Height Z–score; MUAC, mid-upper arm circumference; HAZ, Height-for-Age Z-score; cm, centimetre; kg, kilogrammes; mm, millimetres; sd, standard deviation*.

a *Difference-in-differences analysis*;

b *Linear regression analysis (β coefficient)*,

c *Logistic regression analysis with adjusted odds ratio*.

##### Effect on Health Clinic Visits

Among the three studies that evaluated the impact of CCT on health facility utilisation, two reported a positive effect among beneficiaries (Table 5). Children who benefited from the TASAF programme in Tanzania had significantly more routine clinic visits than controls, although this effect was not significant after 2.5 years of the programme (30).

**Table 5:** Effect of CCT on clinic visits.

**References**	**Study Design**	**Age (months)**	**Outcome**	**Baseline (CCT)**	**Baseline (control)**	**Effect size**
Akresh et al. (29)	Factorial cluster randomised design	0–59	Mean number of preventive health visits in past year	1.03		0.43 (*p* ≤ 0.001)^a^
Adubra et al. (26)	Cluster randomised design	12–42	More than half routine visits (%)	29.0	36.5	3.07 (95% CI 0.93, 10.17)^b^
			One or more routine visits (%)	43.6	44.2	1.36 (95% CI 0.69, 2.70)*^b^*
Evans and Hausladen (30)	Cluster randomised design	0–24	Mean number of health visits in past year	9.2 (combined)		−2.71 (0.1 > *p* > 0.05)^a^^¶^

a *β coefficients from linear models*,

b *Adjusted odds ratio from logistic regression*,

¶ *Presents 95% confidence interval*.

##### Effect on Immunisation Rates

The three studies that assessed the impact of CCTs on the proportion of children vaccinated showed no difference between the CCT beneficiaries and the control groups (Table 6). Beneficiaries of the Manicaland HIV/STD prevention project cash transfers experienced a higher increase in immunisation rates from baseline among beneficiaries (23). Beneficiaries of the SNACK-CNA and SURE-P cash transfers were not more likely to receive immunisations than those who did not (26, 28).

**Table 6:** Effect of CCT on immunisation rates.

**References**	**Study design**	**Age**	**Outcome**	**Baseline (CCT)**	**Baseline (control)**	**Effect size**
Robertson et al. (23)	Cluster randomised design	0–59 months	Complete immunisation records (%)	66.0	66.0	1.9% (95% CI−4.9, 8.8)^a^^¶^
Adubra et al. (26)	Cluster randomised design	12–42 months	Complete immunisation records (%)	82.6	80.1	1.32 (95% CI 0.71, 2.48)^b^^¶^
Okoli et al. (28)	Cluster randomised design	0–6 weeks	Infants vaccinated per 100,000 population	Not reported	Not reported	1.15 (*p* = 0.92)^c^^€^

a *Difference-in-differences analysis*,

b *Adjusted odds ratio from logistic regression model*,

c *βcoefficient from segmented linear regression model for the change in level*,

¶ *Reports 95% confidence interval*,

€ *Reports p-values, CI, confidence interval*.

##### Effect on Utilisation of Specific Health Interventions

The randomised trial by Yotebieng et al. reported no significant difference in proportion of children receiving early infant diagnosis (EID) for HIV (31).

##### Effect on Illness Report

None of the studies reported a significant difference in the frequency of illness reported between CCT recipients and control groups (Table 7). None of the included studies reported on specific childhood illnesses, and all based their measurement on caregivers recall (25, 26, 30).

**Table 7:** Effect of CCT on health status as measured by illness report.

**References**	**Study Design**	**Age (months)**	**Outcome**	**Baseline (CCT)**	**Baseline (control)**	**Effect size (*p*-value or 95% CI)**
Bliss et al. (25)	Quasi-Experimental design	6–24	Mean number of preventive health visits in past year	99	91	+7 (0.17)^a^
Adubra et al. (26)	Cluster randomised design	12–42	Reported ill in the past 15 days (%)	19.9	23.0	1.02 (0.58, 1.08)^b^^¶^
Evans and Hausladen (30)	Cluster randomised design	0–59	Reported ill in the past 4 weeks (%)	75 (combined)		−0.10 (>0.1)^c^
			Number of sick days in past 4 weeks	1.05 (combined)		−0.70 (0.1 > *p* > 0.05)^c^

a *Difference-in-differences*,

b *Adjusted odds ratio from logistic regression*,

c *β coefficient from linear regression*,

¶ *Presents 95% confidence interval, CI, confidence interval*.

#### Thematic Synthesis Exploring Underlying Mechanisms of CCT Impact on Child Health

##### The Incentive Value of the Cash Transfers

The SNACK-CNA pilot project reported that overall, the cash transfer was not considered to significantly influence the decision to visit a health facility. Furthermore, the amount provided in transfers was thought to be insufficient to cover the cost of seeking health care, thereby constraining the incentive value of the transfer (27).

##### How Households Used the Extra Income From the CCT

Responding caregivers in the LEAP pilot in Ghana indicated that food purchase was one of the main uses of the cash benefits. Respondents suggested that the cash transfer increased household income and improved dietary intake. The cash transfers were also used to access health services, as commented by respondents (32). Mothers in the SNACK-CNA pilot likewise reported using the extra income to buy food (and clothes) and to cover health expenses (26, 27).

#### Additional Synthesis by Subgroup

##### Effect of CCT by Level of Household Poverty

The study of Evans et al. (28) and of Akresh et al. (35) report that included subgroup analysis by household poverty level showed no association between the level of household poverty and the effect of CCT on health service utilisation.

##### Effect of CCT by Child Sex

Akresh et al. report a significantly higher number of routine clinical visits per year among female beneficiaries that was not demonstrated in male counterparts (29). Evans et al., however, showed a significantly lower number of yearly clinic visits among female children in the CCTs group; among male children, the yearly clinic visit did not differ significantly between the groups (30).

#### Risk of Bias Across Studies

We did not formally assess the risk of publication bias with a funnel plot due to the small number and the diversity of study designs among the included studies. It was our assessment that a funnel plot may indeed produce misleading results due to this diversity between methodology in the included records (35).

## Discussion

### Summary of Findings

This review aimed to investigate the effect of CCT on utilisation of health services, and on health status. Based on the programme impact theory, we hypothesised that CCT will have a positive impact on child health by improving utilisation of health services and improved nutrition. An important coincidental finding from this review is the paucity of impact studies in this area, and the lack of uniformity in how CCTs are implemented and evaluated. Overall, this review found that there is no evidence that demonstrates a positive effect of CCT on the illness among children in sub-Saharan Africa. The evidence of improved health service use and nutritional status is inconsistent between studies. Despite indications that the extra household income from CCT is mostly used for improving the household diet (25–27, 34), this has not consistently translated into better nutrition for children in SSA. Interestingly, a positive nutritional impact was observed when cash transfers were given to households experiencing sudden and profound food insecurity, as shown in the emergency programme in Niger (25). The evidence of an impact on routine clinic visits was inconsistent across included studies. Furthermore, in one study, an initial observed impact was not sustained on the long term (30). No positive effect on immunisation uptake was demonstrated across all three studies exploring this outcome (23, 26, 28). Overall, we show that CCTs can help remove some of the financial barriers to health service and improved nutrition, but this is not a consistent observation in the studies we reviewed.

While our review suggests that CCT can improve household and childhood diet quality by augmenting household income, there is inconsistent evidence for an association between CCT and improved childhood nutrition based on anthropometric measurement. This is similar to findings from reviews of CCTs in Latin America, which showed a variable impact on anthropometry despite consistent evidence of improved diet among beneficiaries (2, 36). Some programmes showed a positive impact on anthropometry only in younger children (under 24 months). Other programmes like *Programa de Asignación familia* in Honduras and *Familias en Acción* in Colombia did not have a significant impact on anthropometric measures. Many of the programmes in Latin America that did show a positive impact included food fortification and health education as components. A health education component was only included in the emergency CCT programme in Niger (25). Inconsistent effects on health service utilisation have been reported in other reviews as well (1). The current body of evidence suggests that CCTs may only result in an increase in health service utilisation in the short term with the incentive value of the transferred cash diminishing over time. This might explain the observation in the TASAF programme in Tanzania, where a significant increase in clinic visits was seen at 1.5 years after initiation, which disappeared afterwards (30). The same was observed in the PROGRESA programme, where the impact of the programme on preventive health service utilisation was not significant after 8 months (2).

This review is the first to report no positive impact of CCTs on health of children in sub-Saharan Africa. The evidence from other parts of the world has been mixed but overall positive. Findings from PROGRESA in Mexico and *Familias en Acción* in Colombia both show that child beneficiaries were less likely to report illness (1, 2). One possible reason for this difference in observed impact may be due to weaker health systems in sub-Saharan Africa. Also, the conditions were considered “soft” in many programmes in Africa, meaning there were no penalties for not meeting programme requirements.

### Strengths and Limitations of the Review

#### Strengths

This review combines information from multiple sources, across studies and between disciplines, in an attempt to synthesise the evidence about the impact of CCTs in sub-Saharan Africa. To our knowledge, this is one of the first reviews that has focused solely on this region. One important strength of the review lies in the application of a programme impact pathway to guide the review process, which allows for a more in-depth assessment of the impact of CCT. Also, checking for evidence along pathways allowed us to assess causal linkages between components of the intervention, intermediate outcomes, and overall health outcomes.

#### Methodological Limitations of the Included Studies

An important limitation arises from the internal validity of the included records. As reported in the results section of this review, all the quantitative reports had at least one major quality concern. The nature of the intervention makes blinding of beneficiaries impossible; therefore, response bias is a concern in all evaluations of CCT. Blinding of outcome assessors where possible could mitigate the risk of observer bias; this however was not implemented in any of the studies. Observer bias may result in an overestimation of the positive impact of the intervention. However, it is also possible that beneficiaries reported worse outcomes in the hopes that they will receive larger cash benefits. The latter possibility could result in a bias toward finding no impact of CCTs among beneficiaries.

The risk of confounding in most of the included studies was limited as they applied randomisation. However, two studies used a quasi-experimental design which is vulnerable for confounding. None of the reports on cluster randomised studies provided adequate justification for sample size estimation. Also, no justification was given for the number of clusters included in the cluster randomised studies. Finally, some evaluations made multiple comparisons. This can lead to the so-called multiple comparison problem, where spurious associations are identified purely by chance.

#### Limitations of the Review

Other potential limitations of this review include bias due to selective publication and/or differential outcome reporting (reporting bias). Our search strategy was extensive and included grey literature–inclusion of grey literature is expected to reduce reporting and publication bias. On the other hand, most of the grey literature we identified originated from the websites of organisations that are engaged in CCT implementation. These records may, therefore, over-report findings of positive impact. We were unable to formally assess the risk of publication bias with a funnel plot due to the small number, and the large diversity of studies reporting on each outcome (35).

The review would have benefitted from a meta-analysis to produce pooled estimates of effect. However, this was deemed inappropriate in this review due to the observed variability in the included studies. The study designs, context, design of the conditional cash transfer programme and outcome measurement differed between the included studies.

### Interpretation of the Findings

There are multiple possible explanations for the inconclusive evidence around CCT and child health; these may include programme design and implementation fidelity, contextual factors and potential unwanted effects. Very importantly, CCTs likely address only a limited number of the myriad of interlinked factors driving poor child health (37). It is therefore probable, that even in the presence of robust and well-implemented CCT programmes, other factors may undermine the impact. Although many of the intervening determinants of health are influenced by socioeconomic factors (37, 38), it is unlikely that CCT programmes have an impact on the short-term socioeconomic status of household, and long-term socioeconomic benefits have not been established.

In many of the studies in this review, the conditions required for cash transfers were not strictly imposed (described as soft conditions). The soft nature of the conditions could explain why an increase in routine clinic attendance was not observed in some programmes despite this being a requirement for cash transfers (26, 30). Secondly, inefficient targeting may result in the programme missing out on the most vulnerable children within the community. Ineffective targeting may also be lead to a reduction in overall effect of CCT programmes.

Many of the rural populations in SSA where CCTs have been implemented experience multi-dimensional barriers to essential health services. The size and nature of these barriers may influence the impact of CCT, with greater impact where financial constraints are responsible for poor uptake (1). Conversely, where cultural and religious beliefs result in poor health services utilisation, conditional financial incentives may be ineffective. Furthermore, most of the reviewed CCTs were implemented in countries with poor health system indicators—e.g., measured by health worker density (Supplementary Table 1). In these cases, much of the poor access is driven by supply-side constraints and CCTs are unlikely to address health access problems. Indeed, some experts suggests that supply-side intervention, including health-system improvements, are required for the success of any CCT programme (1).

Unwanted effects have been reported in relation to CCTs, and these effects may limit impact on beneficiaries or have a detrimental effect on non-beneficiaries. While unwanted effects were not discussed in any of the programmes included in this review, they remain a possibility. For instance, CCTs have been known to result in an increase in food prices in communities (39). This may have constrained the impact of the intervention on childhood nutrition or even led to poorer nutrition in non-beneficiaries. As a final point, it is possible that the extra income may have been used for other household purposes. For instance, there was reported increased spending on clothing for adult females in household who benefited from the TASAF CCT programme.

## Conclusion and Recommendations

This review highlights the paucity of rigorous studies reporting on the impact of CCTs on child health and health service utilisation in sub-Sahara Africa. There is inconclusive evidence that these interventions improve access to health services for children in sub-Saharan Africa. These findings contrast with evidence from other regions of the world where impact on health status and access to health services has been more positive.

### Implications for Practise

Several important considerations became apparent during the conduct of this review, which should inform future implementation of CCTs within SSA. Firstly, it is important that the design of CCTs—and indeed any type of intervention aimed at improving health access—is based on sound understanding of the context-specific drivers of poor health access (demand vs. supply constraint). Where the supply of health services is poor (no availability), interventions like CCTs that aim to improve demand are unlikely to have significant impact.

Due consideration should be given to the amount of cash that is likely to have a positive incentive effect on recipients. We see that most of the CCT programmes with small cash transfer did not have a positive effect on health service use. This then raises the important consideration of the cost of these programmes and their sustainability. Evidence suggests that CCT programmes are associated with large administrative costs related to targeting and monitoring of participant's compliance to conditions. This high running cost may make these interventions unsustainable in the long term (1).

Furthermore, in the design of CCTs targeting children, it appears that certain components, such as food fortification, and health and nutrition education are particularly relevant. It is therefore important that these components are considered in any such interventions aimed at improving nutrition and health of children.

Finally, the conditional cash transfers have been criticised for imposing conditions on cash transfers, infringing on individual freedoms and decision-making capacity, as well as enshrining structural power imbalance (within communities and between provider and beneficiaries) (40). Some authors have cited the burden on beneficiaries, unpredictable attainability of the conditions, receptiveness of beneficiaries, and the potential for negative effect as potential problems with CCT programmes (41). For instance, beneficiaries of CCT may not be able to meet conditions due to wider contextual factors, such as distance to facility, poor roads, or even a health system that is not patient-centred. This further underlies the importance of a context-specific approach in designing CCT programmes. Significant background knowledge of the local context is required in the design of ethical CCT programmes.

### Implications for Future Research

The first area for further research involves trying to understand the influence of contextual factors on the impact of CCTs. The current published studies are inadequate in achieving a full understanding of how, where and for whom CCT programs have benefits. A theory-driven rather than method-driven approach is necessary, especially one that situates the programmes within a wider system and environment. This is particularly relevant for interventions designed to address complex problems (39).

A second future research area is to separate and understand the different components of the CCT strategy. Gaps remain in the literature on the mechanisms by which these interventions work, the pathways by which they work and the influence of context. This will require the development of detailed programme impact frameworks based on sound theory. These frameworks will help in the design of evaluations that could be better suited to explain the key components of the CCT strategy. CCTs by definition are complex and therefore any evaluation of their impact should take into consideration this complexity (40).

There is also the need to compare the impact of CCTs with other service delivery interventions on improving health access for children. This will hopefully help inform policy choices on the most effective approach to improving child health and access to health services. In addition, the cost of these interventions needs to be carefully documented and evaluated to help guide policy decisions.

## Data Availability Statement

The original contributions presented in the study are included in the article/Supplementary Material, further inquiries can be directed to the corresponding author/s.

## Author Contributions

CO contributed to conceptualisation, literature search, study selection, quality assessment of eligible studies, analysis/synthesis, and writing of initial manuscript. KV and BM contributed to conceptualisation, study selection, analysis/synthesis, and review of manuscript. All authors contributed to the article and approved the submitted version.

## Conflict of Interest

The authors declare that the research was conducted in the absence of any commercial or financial relationships that could be construed as a potential conflict of interest.
