# Supplementary material for: Systematic Review on the Impact of Conditional Cash Transfers on Child Health Service Utilisation and Child Health in Sub-Saharan Africa
Source: Front Public Health. 2021 Jul 14;9:643621. doi: 10.3389/fpubh.2021.643621 (PMC8316722; doi:10.3389/fpubh.2021.643621)
Supplement: Supplementary file 1 [file Table_1.DOCX]

**Conditional cash transfers**

**Health service utilization**

1. Immunization
2. Growth monitoring
3. Micronutrient supplement

Program conditions

**Incentivization**

Use to access or pay for health services

**Mother empowered**

Improved nutritional state

Improved diet for child

Purchase healthier foods

**higher income for food**

**Improved health**

Cash provided to mother

Health & Nutrition education

**Context**

Figure 1. Mechanisms by which CCT might affect health service utilization and health outcomes in under-fives.

We approached this review with a programme impact theory developed on the basis of a model by Leroy and colleagues and elements from the work of de Groot and colleagues. This framework describes the pathway(s) by which component interventions of a programme are theorised to produce their desired outcomes. Fundamental to this is the understanding that complex interventions usually have intermediate outcome(s) which are necessary to achieve the eventual outcome. In programmes with multiple interventions, such as CCTs, exploring these intervention-outcome relationships should consider intermediate outcomes along these pathways. For instance, CCTs may improve child health outcomes (final outcome) through multiple intermediate outcomes (e.g. improved health service utilisation, increase use of preventive services etc.). We expound on the proposed impact framework as it relates to health outcomes

Cash transfers to household may increase household income, and if this increase in income is deployed toward improved food quality that household and children consume, this results in better nutritional status (income-diet-nutrition pathway). This improved diet may subsequently improve overall health by improved nutritional state and immunity. However, the transferred cash may be used for other commodities and not result in better dietary quality and quantity (competing commodity). Also important to note, an increase in household income may not translate to healthier diets if the mothers or caregivers do not have the necessary nutritional knowledge (caregiver nutrition knowledge). In addition, nutrition training may result in mothers or caregivers making healthier dietary choices for children.

Another potential consequence of an increase in household income is an increase access to health services. The extra income may help households overcome the geographic and financial barriers to health service use (income-health service utilisation pathway). Also, CCTs as a demand side intervention is unlikely to work in situations where the health system (supply side) is weak. Conditions linked with the CCTs may also improve health service utilisation and health via more direct pathways. The conditions mean that mothers are obliged to bring children to health facilities for preventive and curative health services. This could result in increase in immunisation rates, frequency of growth monitoring and uptake of deworming interventions.

Most cash transfer programs are designed with mothers as the recipient of the cash; this is theorised to give mothers greater control over how the use of the funds. This greater empowerment is likely to increase the likelihood that cash transfers go toward the child’s diet (mother empowerment).

Evaluations of CCTs have also shown that recipient mothers feel a greater sense of empowerment and feel they have a greater role in decision making within the household. Furthermore, cash transfers may serve as a direct incentive for households to use health services and to adopt healthy behaviour (incentive effect). It is widely held that incentives, financial or otherwise, can influence human behaviour and the choices people make. It is also understood that incentives are more effective for infrequent behaviours (e.g. attending immunisation) than for continuous behaviours. The influence of the incentive is likely to depend on the size of the transfer, the influence of the barriers to utilisation, and the level of poverty of recipients. For example, a study on CCTs as a means to increase Human immunodeficiency virus (HIV) result collection showed that poorer individuals were more sensitive to cash transfers and larger transfers were associated with greater compliance with programme requirements. However, incentives alone may not be adequate to overcome the barriers to access. For instance, cash incentives may not cover expense or overcome geographical barriers, and may not be adequate to overcome cultural barriers to access.

We go further in our theory by situating the cash transfer interventions within a wider context or a programme environment. This include programme design, how recipients interface with the programme, degree of household vulnerability and effectiveness of targeting, duration of participation, and adequacy of the supply-side all may modify the effects of CCT on health and nutrition.
